# Perceiving direction of deformation-based motion

**DOI:** 10.1177/20416695251364725

**Published:** 2025-08-25

**Authors:** Takahiro Kawabe

**Affiliations:** Communication Science Laboratories, NTT Inc., Japan

**Keywords:** image deformation, deformation-based motion, spatial frequency of deformation, displacement speed

## Abstract

In dynamic visual scenes, many materials—including cloth, jelly-like bodies, and flowing liquids—undergo non-rigid deformations that convey information about their physical state. Among such cues, we focus on deformation-based motion—defined as the spatial shifts of image deformation. Studying deformation-based motion is essential because it lies at the intersection of motion perception and material perception. This study examines how two fundamental properties—spatial frequency and displacement speed—jointly shape the perception of deformation-based motion. We focused on these parameters because, in luminance-based motion perception, spatial frequency and displacement speed have been shown to critically influence motion sensitivity. Across three experiments using sequentially deformed 1/f noise images as a neutral background, we systematically manipulated the spatial frequency components of the deformation and the speed at which these deformations were displaced. Results showed that direction discrimination performance was strongly modulated by the interaction between spatial frequency and displacement speed. Suppressing local deformation cues improved discrimination at low frequencies, suggesting that local signals may interfere with global motion inference. These findings reveal how the spatial structure and dynamics of image deformation constrain motion perception and provide insights into how the brain interprets dynamic visual information from non-rigid materials.

## How to cite this article

Takahiro Kawabe (2025). Perceiving direction of deformation-based motion. *i-Perception*, 16(4), 1–12.

## Introduction

Various materials undergo nonlinear deformations. These materials dynamically change shape due to continuous internal or external forces. For example, a fabric like a flag flutters in the wind. By observing this fluttering motion, we can infer that wind is blowing and also perceive the texture of the fabric (Bi et al., 2018, 2019; Bi & Xiao, 2016; Bouman et al., 2013; Wijntjes et al., 2019). Similarly, because many organisms have bodies composed partly of elastic materials, their bodies dynamically deform as they move (Kawabe, 2017; Michotte, 1963). In the case of liquids, the liquid itself deforms as it flows (Kawabe, Maruya, Fleming et al., 2015), and if the liquid is transparent, refraction causes the background image to become distorted. This distortion dynamically changes over time. Such dynamic deformations likely serve as important cues for perceiving the state of external materials (Kawabe, 2018; Kawabe, Maruya and Nishida, 2015; Kawabe & Kogovšek, 2017).

On the other hand, although we can perceive a variety of dynamic deformations, the characteristics of the information processing involved in perceiving the direction of deformation-based motion remain poorly understood. Hereinafter, “deformation-based motion” refers to the linear spatial shift of image deformation driven by the spatial displacement of deformation maps, whereas “local deformation” refers to image deformation occurring within localized regions of an image. This study focuses on how the spatial frequency of image deformation influences the perception of deformation-based motion. When the spatial frequency of the dynamic deformation is low, it tends to be perceived as the deformation of the material itself (Kawabe & Sawayama, 2020). In contrast, when the spatial frequency is high, the deformation is perceived as if there were a transparent medium in front of the material, with the background image dynamically deforming behind it (Kawabe, Maruya & Nishida, 2015) . While the perceptual appearance varies with the spatial frequency of the deformation, we hypothesize that the perception of deformation-based motion—a fundamental characteristic of dynamic image deformation—relies on a shared mechanism of information processing.

To investigate these processing characteristics, this study examines the interaction between the spatial frequency of dynamic image deformation and a displacement speed of a spatial deformation pattern. Previous studies using luminance patterns have shown that the detection threshold for motion depends on the spatial frequency of the pattern (Boulton & Baker, 1991). Based on this, we hypothesize that a similar dependency on the interaction between spatial frequency of image deformation and displacement speed may also be observed in the perception of direction of deformation-based motion.

Investigating the perception of deformation motion direction is in general beneficial for understanding the mechanism for motion perception mechanism. While many previous studies (Adelson & Bergen, 1985; Viva & Morrone, 1998) have explored how the visual system detects motion direction, most have focused on rigid luminance motion, in which motion signals are spatially uniform and/or luminance-defined. In contrast, the local motion vectors in deformation motion exhibits spatially non-uniform motion signals. For example, it is known that transparent liquid flows exhibit spatiotemporally lowpass patterns of local motion vectors (e.g., Kawabe, Maruya, Fleming et al., 2015, Figure 2A). This poses a significant challenge to conventional motion detection models, which generally assume spatially coherent motion patterns as inputs. As such, it remains an open question how the visual system integrates heterogeneous motion signals to infer a coherent global direction in non-rigid motion.

The present study investigated the spatial characteristics of direction perception in deformation-based motion. We hypothesized that a similar frequency-dependent effect would emerge in deformation-based motion: specifically, that discrimination performance would decline when the displacement speed exceeds a certain proportion of the wavelength defined by the deformation's spatial frequency. Experiment 1 tested this hypothesis by examining the interaction between the cut-off spatial frequency of the deformation and the displacement speed. Participants reported the perceived direction of deformation-based motion, while the spatial frequency bandwidth and displacement speed were systematically manipulated. Experiment 2 extended this investigation by using deformation maps based on band-pass noise stimuli to further examine how specific spatial frequency bands interact with displacement speeds in shaping motion direction perception. Experiment 3 addressed the role of local deformation in this perceptual process. We examined whether local motion cues—potentially acting as noises to the perception of deformation-based motion—bias participants’ perception of motion direction. Together, the results suggest that irrespective of the spatial frequency of image deformation, the detection of unidirectional motion signals plays a crucial role in perceiving the direction of deformation-based motion. We further discuss the possible role of such signals in perceptual mechanisms underlying transparency perception.

## Method

### Participants

In Experiment 1, 18 people (8 females) participated. Their mean age and standard deviation are 27.61 and 9.21, respectively. In Experiments 2, 16 people (8 female) participated. Eight of the people had also participated in Experiment 1. Their mean age and standard deviation are 30.25 and 11.22, respectively. In Experiment 3, the people, who had participated in Experiment 2, also participated. The sample size was determined using MorePower 6.0.4 (Campbell & Thompson, 2012), based on 4 × 7 within-participants factor models, a significance level of α = 0.05, desired statistical power of 0.90, and an expected effect size of η² = 0.10. Because no directly comparable prior studies were available, we assumed a medium-to-large effect size (η² = 0.10), following the conventional conversion from Cohen's *f*. The analysis indicated that a minimum of 14 participants would be required to achieve reliable statistical results. Participants were recruited through a human resource agency in Japan and received monetary compensation based on criteria determined by the agency, which were not disclosed to the researchers. All participants were naive to the specific purposes of the experiments. Ethical approval for the study was obtained from the Ethics Committee of NTT Communication Science Laboratories (approval number: R06-013). The experiments adhered to the principles of the 2013 Declaration of Helsinki. Written informed consent was obtained from all participants prior to their participation.

### Apparatus

Stimuli were presented on an LCD monitor (Display++, Cambridge Research Systems Inc., USA). The monitor's luminance output was linearly calibrated using a luminance meter (LS-150, Konica Minolta Inc., Japan), with a luminance range of 0–144 cd/m². A Mac Pro computer (Apple Inc., USA) was used to control stimulus presentation and data collection. The experimental scripts were written by using PsychoPy (Peirce et al., 2019). The observation distance was 0.573 m.

### Stimuli

**Figure 1.**
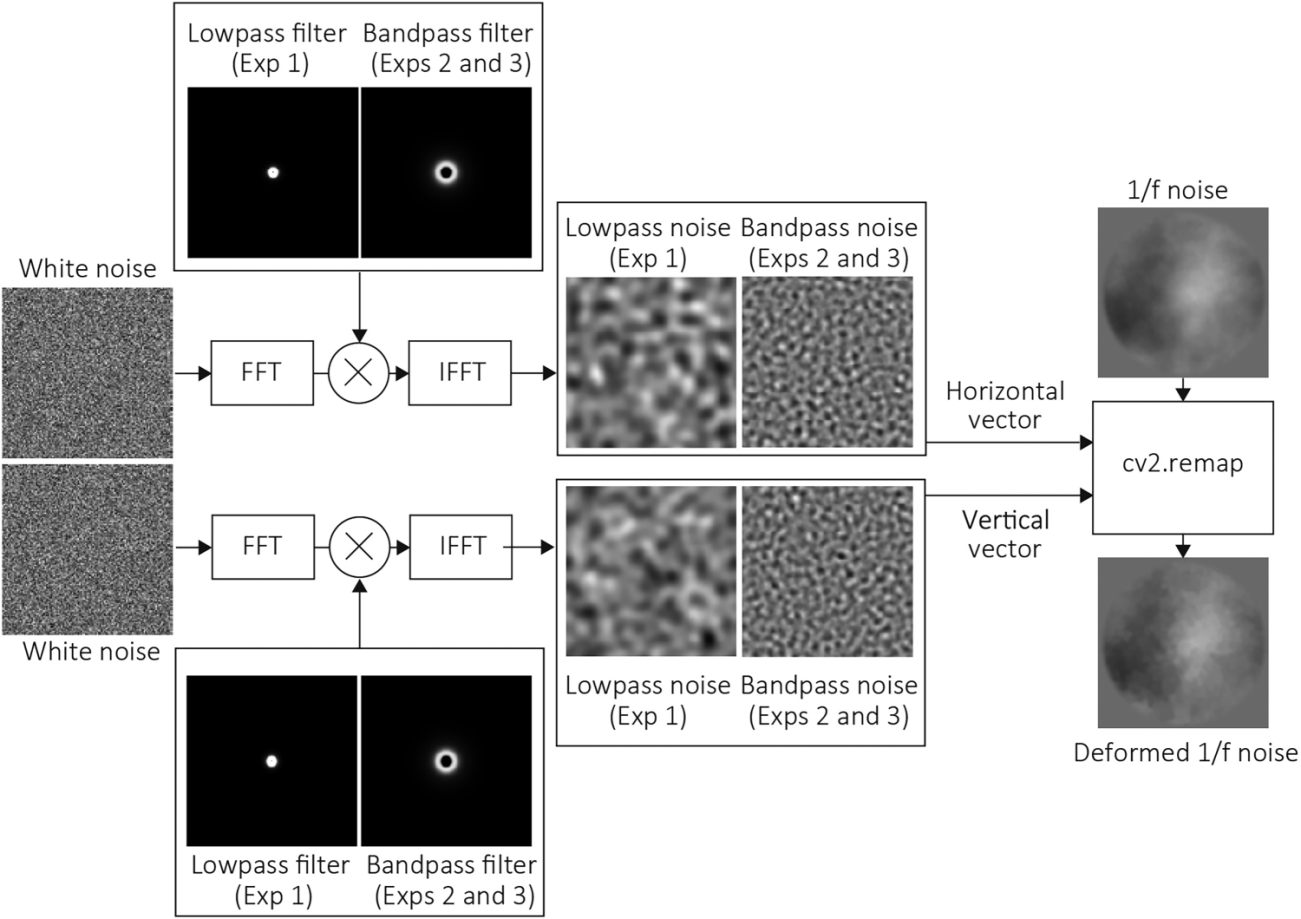
Schematic Depiction of the Stimulus Generation Process. Two Sets of White Noise were Lowpass-Filtered (in Experiment 1) or Bandpass-Filtered (in Experiments 2 and 3) and Used as Horizontal and Vertical Vectors to Deform 1/f Noise as the Background. The Deformed 1/f Noise Served as One of the Video Frames in a Stimulus Clip. The Deformation Displacement in the Clip was Generated by Shifting the White Noise.

Stimuli were two-dimensional 1/f noise images that were sequentially deformed. (See Figure 1 for details of stimulus generation. Movie 1 shows example stimulus clips containing rightward deformation-defined motion, with the cut-off frequency and deformation magnitude indicated in the top-left corner of each clip.). Each stimulus measured 256 × 256 pixels and was presented for 0.5 s at a frame rate of 15 Hz. For each trial, a new 1/f noise image was generated and deformed as described below. We chose the 1/f noise as a background image based on the previous study (Kawabe, Maruya & Nishida, 2015). To deform the image, two sets of two-dimensional white noise were generated as deformation maps: one for horizontal deformation and the other for vertical deformation. Each set consisted of 15 frames, with each frame measuring 256 × 256 pixels. The white noise was filtered either using a low-pass filter (Experiment 1) or a band-pass filter (Experiments 2 and 3). The filters were created based on a two-dimensional Butterworth filter with an order of 14. For low-pass filtering, the cut-off spatial frequency was set at one of four levels: 4, 8, 16, or 32 cycles per image (cpi), corresponding to 0.4, 0.8, 1.6, and 3.2 cycles per degree (cpd). The minimum wavelengths for these levels were 2.5, 1.25, 0.63, and 0.31 degrees, respectively. For band-pass filtering in Experiment 2, the range was set to one of four octave bands: [2–4, 4–8, 8–16, and 16–32 cpi], corresponding to [0.2–0.4, 0.4–0.8, 0.8–1.6, and 1.6–3.2 cpd]. Movie 2 shows example stimulus clips containing rightward deformation-defined motion, with the higher value of octave bands and deformation magnitude indicated in the top-left corner of each clip. The filtered noise values were standardized to a range of −12 to 12 and were used as deformation magnitudes. The deformation was implemented using OpenCV's cv.remap function, which applied the deformation maps to the 1/f noise. For each frame, the white noise was spatially displaced in one of four directions (upward, downward, leftward, or rightward) with magnitudes of 2, 4, 8, 16, 32, 64, or 128 pixels per frame, corresponding to 0.07, 0.15, 0.31, 0.62, 1.25, 2.5, and 5 degrees per frame, respectively. We expect that direction discrimination performance decreases with increasing displacement speed and approaches chance level when the displacement is 128 pixels (5 degrees) per frame. The deformed 1/f noise was then processed with a two-dimensional Tukey window with a diameter of 9 degrees. The final stimuli were presented to participants at a frame rate of 30 Hz for 0.5 s. In Experiment 3, we aimed to eliminate the influence of image deformation on direction discrimination. To achieve this, the original (intact) 1/f noise was subtracted from the deformed stimuli, and the resulting subtraction images were overlaid on a uniform neutral gray background before being presented to participants. Because the background images used in this study consisted of 1/f noise and the amount of deformation between frames was relatively small, subtracting the original image from the deformed image did not result in large changes in luminance. Therefore, no additional image processing, such as clipping, was necessary. Movie 3 shows example stimulus clips containing rightward deformation-defined motion, with the higher value of octave bands and deformation magnitude indicated in the top-left corner of each clip.

### Procedure

Each participant was tested individually in a dimly lit experimental room. A head and chin rest were used to stabilize the participant's head and ensure a consistent visual field. To initiate each trial, participants pressed the space bar. Following this, sequences of deformed 1/f noise images were presented for 0.5 s. After the sequence disappeared, participants reported the perceived direction of deformation-based motion using a four-alternative forced-choice task. They indicated whether the motion appeared to be upward, downward, leftward, or rightward by pressing the corresponding keys on the keyboard. Each participant completed four sessions, with each session consisting of 140 trials. The trials included seven levels of displacement speed, four motion directions, and five repetitions per condition. The trial order within each session was randomized. In Experiment 1, one cut-off frequency condition was tested per session. In Experiments 2 and 3, one frequency range condition was tested per session. The order of sessions was randomized within each experiment. Each participant completed all four sessions in approximately one hour, including breaks.

### Statistics

The proportion of correct responses for discriminating the direction of deformation-based motion was calculated for each experimental condition. In Experiment 1, a two-way repeated-measures ANOVA was performed with cut-off frequency and displacement speed as within-participant factors. In Experiments 2 and 3, a two-way repeated-measures ANOVA was conducted with frequency bands and displacement speed as within-participant factors. Due to violations of the sphericity assumption, Greenhouse-Geisser corrected *p*-values were used for interpreting the results. For post-hoc tests, Bonferroni-corrected *p*-values were applied. Effect sizes were reported using eta-squared (η²).

## Results and Discussion

Supplemental Data 1 provides the raw data for Experiments 1, 2, and 3. Supplemental Data 2, 3, and 4 present the results of OLSs for Experiments 1, 2, and 3, respectively. Hereinafter, we summarize the significant findings of the experiments. For further details, please refer to the Supplemental data.

### Experiment 1

**Figure 2.**
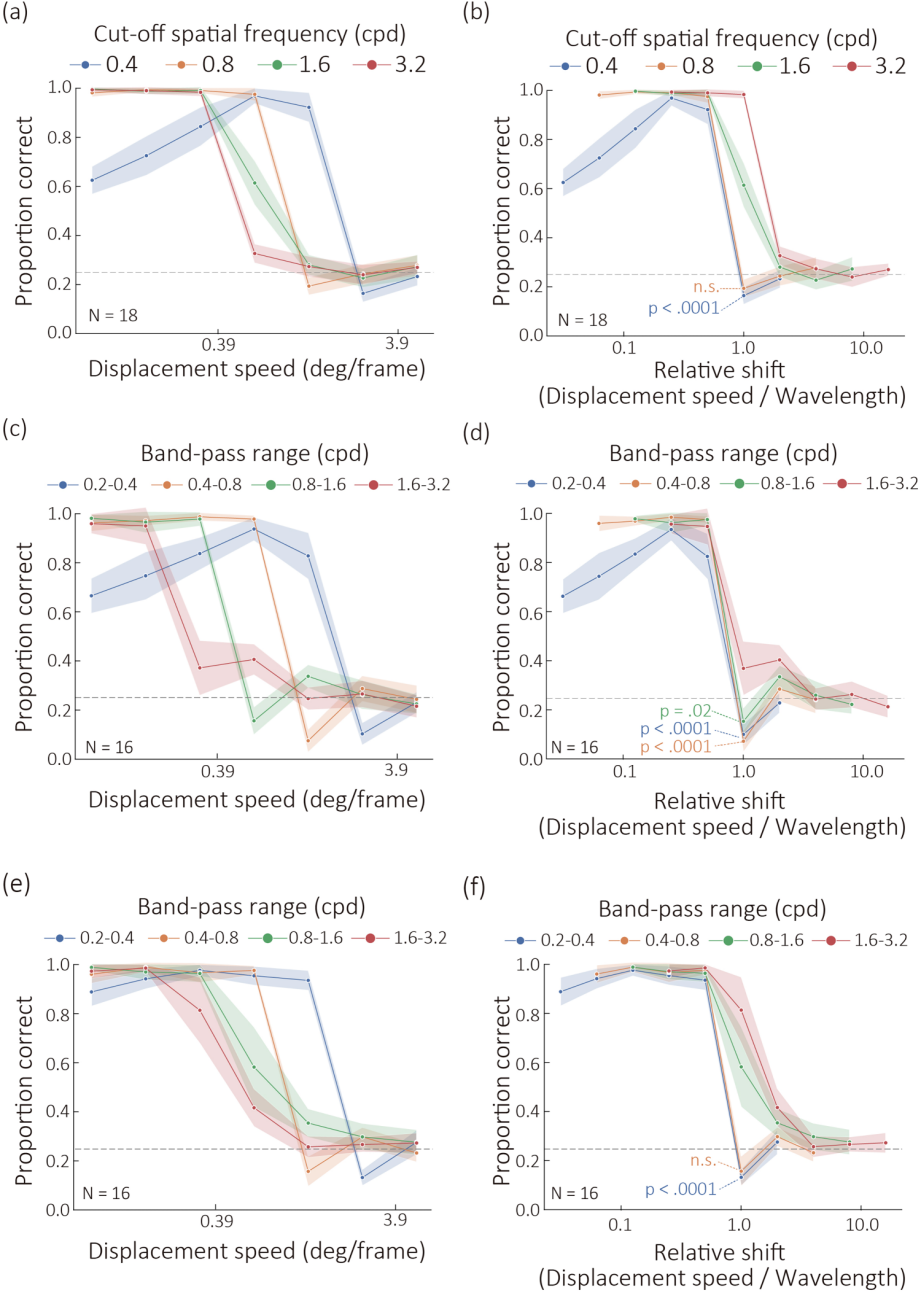
Experimental Results from (a) Experiment 1, (c) Experiment 2, and (e) Experiment 3, with the Results Replotted as a Function of Relative Shift in (b), (d), and (f), Respectively. Stripes Denote 95% Confidence Intervals.

Figure 2a shows proportion correct for the direction discrimination as a function of displacement speeds for each cut-off frequency condition. We conducted a two-way ANOVA and acknowledged that both the main effect of the cut-off frequency (*F* (3,42) = 13.682, *p_gg_corrected_* < .0001, *η^2^* = 0.14, *ε* = 0.70) and the main effect of the displacement speed (*F* (6,84) = 1061.001, *p_gg_corrected_* < .0001, *η^2^* = 0.938, *ε* = 0.69) were significant. The interaction between the two factors was significant (*F* (18, 252) = 121.913, *p_gg_corrected_* < .0001, *η^2^* = 0.833, *ε* = 0.22). For all conditions of cut-off frequencies, the simple main effect of the displacement speed was significant (*p* < .0001). The simple main effect of the cut-off frequencies was significant (*p* < .005) for all displacement speed, except when the displacement speed was 5 deg. When the cut-off frequency was 0.4 cpd, the performance peaked at the displacement speeds of 0.62 and 1.25 deg. That is, the performance was best at specific displacement speeds. On the other hand, when the cut-off frequencies were 0.8, 1.6, and 3.2 cpd, the performance was better when the displacement speeds were smaller. The results show that discrimination performance as a function of displacement speed strongly depends on the cut-off frequency, suggesting that the mechanism underlying the discrimination of deformation-based motion is tuned to displacement speed in a cut-off-frequency-dependent manner. When the cut-off frequencies were lower, the discrimination performance was higher at larger displacement speeds. Specifically, when the displacement speed was 0.62 deg, the performance was significantly lowest at 3.2 cpd, but it was significantly highest at 0.4 and 0.8 cpd. These results are consistent with the previous study (Boulton & Baker, 1991) which showed that higher spatial frequencies resulted in a lower maximum displacement for the perception of coherent motion.

Next, we examined the proportion of displacement speed relative to the wavelength, referred to as the “relative shift,” as we hypothesized that analyzing this measure is essential to evaluate whether the characteristics for discerning deformation-based motion direction are consistent across different cut-off frequency conditions. Figure 2b presents the proportion of correct responses as a function of relative shift for each cut-off frequency condition. As the cut-off frequency increased, performance declined at higher relative shifts. These results suggest that the ability to discriminate motion direction is not consistent across cut-off frequencies. One possible explanation for this inconsistency is the use of low-pass filters to generate deformation maps. In higher cut-off frequency conditions, the stimuli contain more broadband frequency components of image deformation, which may contribute to retaining motion direction discrimination even at higher relative shifts.

### Experiment 2

Experiment 2 investigated how performance declines in response to relative shifts when deformation maps are generated using band-pass filters. Instead of cut-off frequencies, we examined the role of frequency bands of band-pass filters in the direction discrimination (see Movie 2 for example stimulus clips containing rightward deformation-defined motion). Specifically, we tested the following four frequency bands: 0.2–0.4, 0.4–0.8, 0.8–1.6, and 1.6–3.2 cpd. Figure 2c shows proportion correct for the direction discrimination as a function of displacement speeds for each frequency band condition. As in Experiment 1, we conducted a two-way ANOVA and acknowledged that both the main effect of the frequency bands (*F* (3,45) = 27.686, *p_gg_corrected_* < .0001, *η_g_^2^* = 0.26, *ε* = 0.77) and the main effect of the displacement speed (*F* (6,90) = 641.944, *p_gg_corrected_* < .0001, *η_g_^2^* = 0.885, *ε* = 0.53) were significant. The interaction between the two factors was significant (*F* (18, 270) = 110.446, *p_gg_corrected_* < .0001, *η_g_^2^* = 0.79, *ε* = 0.26). For all conditions of frequency bands, the simple main effect of the displacement speed was significant (*p* < .0001). The simple main effect of the frequency bands was significant (*p* < .0001) for all displacement speed, except when the displacement speed was 5 deg (*p* = .855). When the frequency band was 0.2–0.4 cpd, performance peaked at a displacement speed of 0.62 degrees. Consistent with Experiment 1, the best performance occurred at specific displacement speeds. In contrast, for frequency bands of 0.4–0.8, 0.8–1.6, and 1.6–3.2 cpd, performance improved as displacement speeds decreased. Figure 2d illustrates the proportion of correct responses as a function of relative shift. Compared to Experiment 1, the variability in performance across frequency bands was reduced. These results suggest that when the frequency band of image deformation is properly controlled, the discrimination of deformation-based motion is processed more consistently across frequency bands.

An unresolved issue is why performance declines when the displacement speed is small under the low spatial frequency band condition (0.2–0.4 cpd). Previous research (Nakayama & Silverman, 1988) has demonstrated that discrimination performance for the translation direction of deformation improves with higher spatial frequencies of deformation. A subsequent computational study further revealed that as the spatial frequency increases, the perception of deformation-based motion transitions from deformation to translation (Kawabe & Sawayama, 2020). Building on these findings, we hypothesize that in the low spatial frequency band, local deformation perception becomes dominant. This dominance reduces the salience of deformation-based motion (i.e., the spatiotemporal shift of local deformation), leading to poorer performance in direction discrimination under low-frequency conditions. To test this hypothesis, we conducted the following experiment.

### Experiment 3

In this experiment, we subtracted an intact (non-deformed) background image from each video frame in the stimulus clips used in Experiment 2. This method effectively attenuated the influence of local deformation in the discrimination task for deformation-based motion (see Movie 3 for example stimulus clips containing rightward deformation-defined motion). Participants performed the same task as in Experiments 1 and 2, and we examined whether this manipulation could improve performance at smaller displacement speeds in the low spatial frequency band of image deformation, where performance had been lower in previous experiments.

Figure 2e shows proportion correct for the direction discrimination as a function of displacement speeds for each frequency band condition. As in Experiment 2, we conducted a two-way ANOVA and acknowledged that both the main effect of the frequency bands (*F* (3,45) = 40.293, *p_gg_corrected_* < .0001, *η_g_^2^* = 0.232, *ε* = 0.56) and the main effect of the displacement speed (*F* (6,90) = 678.307, *p_gg_corrected_* < .0001, *η_g_^2^* = 0.894, *ε* = 0.542) were significant. The interaction between the two factors was significant (*F* (18, 270) = 46.212, *p_gg_corrected_* < .0001, *η_g_^2^* = 0.646, *ε* = 0.135). For all conditions of frequency bands, the simple main effect of the displacement speed was significant (*p* < .0001). The simple main effect of the frequency bands was significant (*p* < .0001) for all displacement speed, except when the displacement speed was 5 deg (*p* = .855). Importantly, in the lowest frequency band condition, the performance at 0.07 deg was statistically comparable to that at 0.15, 0.31, 0.62, and 1.25 deg (*p* > .15). Similarly, the performance at 0.15 deg was comparable to that at 0.31, 0.62, and 1.25 deg (*p* > .12), and the performance at 0.31 deg was comparable to that at 0.62 and 1.25 deg (*p* = 1.00). These results support our hypothesis that discrimination of deformation-based motion direction does not decline with smaller displacement speeds even in the lowest frequency band condition.

Figure 2f shows the proportion of correct responses as a function of the relative shift. Compared to the results from Experiment 2 (Figure 2d), the discrimination performance was less consistent across different frequency band conditions, particularly at larger displacement speeds. We speculate that this inconsistency may be due to the subtraction of the original (intact) background image from the deformed images, which likely reduced interference from local deformations in the discrimination of deformation-based motion. However, the exact source of this interference remains an open question.

### Additional Analysis

In all experiments, the performance dropped below the chance level (0.25). To check whether the performance was significantly lower than the chance level, we conducted Holm–Bonferroni corrected one-sample t-tests for each of the seven frequency bands. To control for the family-wise error rate due to multiple comparisons, *p*-values were adjusted using the Holm–Bonferroni method (Holm, 1979) across the seven tests. The results of the one-sample t-tests are shown in Supplemental Data 5 and partly in Figure 2. Moreover, notations in Figure 2 show the significance of the one-sample t-test when the relative shift is 1. In some conditions, especially when the cut-off spatial frequency was low, the performance was significantly lower than the chance level. As the relative shift increases, the resultant motion signals possibly become ambiguous, and the participants may make use of the direction of motion signals in local deformations to judge stimulus motion directions. Because the local deformations contain motion signal opposite to the deformation-defined motion, the stimulus motion direction might be reported with a bias in an opposite direction to the deformation-defined direction.

## General Discussion

The present study investigated the properties of discriminating the direction of deformation-based motion. Experiments 1 and 2 demonstrated that the spatial frequency components of image deformation are key determinants of direction discrimination in terms of displacement speed. Specifically, lower spatial frequencies of image deformation contributed to maintaining discrimination performance even at larger displacement speeds. Experiment 3 further revealed that attenuating local deformation enhanced discrimination performance, particularly at lower spatial frequencies, suggesting a potential interaction between the processing of deformation-based motion and local deformation signals.

In this study, we would like to discuss its influence of using a 1/f noise as the background. We selected 1/f noise as the image to be deformed because its contrast characteristics are similar to those of natural images (Burton & Moorhead, 1987; Field, 1987) . However, elements like riverbed pebbles or grass create sharp contrast edges in retinal images. Therefore, conducting follow-up experiments with backgrounds that have clear luminance edges would be important for further elucidating the mechanisms of deformation-based motion perception.

It would be beneficial to discuss the ecological validity of the stimuli used in the present study. In this study, we employed dynamically deformed background images generated using low-pass and band-pass deformation maps. In a prior study, Kawabe, Maruya and Nishida (2015) analyzed deformations of background images caused by real liquid flows and found that the spatiotemporal frequency characteristics of such deformations exhibited low-pass properties. The stimuli used in Experiment 1 of the present study were created based on these previous findings. Therefore, it can be said that the stimuli in this study possess a certain degree of ecological validity. However, there remain some aspects that warrant critical consideration. First, it has not been confirmed whether the image deformations obtained by linearly translating low-pass deformation maps in one direction are actually observed in natural liquid flows. Second, as in the analysis by Schlüter and Faul (2019) involving static transparent objects, we did not conduct a detailed manipulation of how background deformations vary as a function of surface geometry or conform to natural phenomena. Third, the relationship between the low spatial frequency of image deformation and the physical deformation of the external object itself was not examined. Taken together, future research should aim to implement more precise stimulus manipulations in order to address these limitations and further enhance ecological validity.

In this study, we investigated the perceived direction of deformation-based motion; however, the direction of such motion does not necessarily correspond to the actual movement direction of a physical object. For example, when a transparent object with a refractive index moves in front of a background, there are physical conditions under which the apparent motion of the background seen through the object may differ from the object's actual direction of movement. Similarly, when a transparent liquid with a complex and temporally varying surface moves, the direction of deformation observed in the image does not always coincide with the actual flow direction of the liquid. Therefore, the findings of this study are limited to the perceived direction of deformation-based motion on the image level, and it should be noted that directly linking these perceptual findings to the motion direction of external physical objects is not straightforward.

The absolute level of discrimination performance may be influenced by the experimental settings. In the present study, the frame rate of the stimulus clips was set to 30 Hz. Thus, for example, with larger displacement speeds, the visual system must resolve greater spatial jumps in image deformation to discern the direction of deformation-based motion. The performance might have been improved if a higher frame rate had been used.

Our results suggest a connection between the detection of motion signals arising from local deformation and those originating from the spatiotemporal displacement of local deformation (deformation-based motion). In Experiment 3, eliminating local deformation enhanced discrimination performance, particularly in the lower spatial frequency bands. Phenomenally, participants could perceive both the direction of deformation-based motion and the local deformation simultaneously in the stimuli used in Experiments 2 and 3. Future studies should investigate how the discrimination of deformation-based motion direction integrates with the phenomenological experience of the stimuli.

Local deformation has been discussed in this study as a factor that hinders the perception of deformation-based motion direction. However, in real-world contexts, the perception of local deformation likely plays an important role in material perception. For example, the degree of local deformation could serve as a cue for estimating the thickness of a transparent medium (Fleming et al., 2011) though the effect of other material cues like specular reflection (Schluter & Faul, 2014) should also be considered. While this study focused exclusively on directional perception, future research could investigate how humans integrate material perception based on local deformation with the directional perception of deformation-based motion in natural environments. Such investigations would provide valuable insights for the perception of materials from image deformation.

## Supplemental Material

**Figure:** Video 1.

**Figure:** Video 2.

**Figure:** Video 3.

sj-xlsx-1-ipe-10.1177_20416695251364725 - Supplemental material for Perceiving direction of deformation-based motion Supplemental material, sj-xlsx-1-ipe-10.1177_20416695251364725 for Perceiving direction of deformation-based motion by Takahiro Kawabe in i-Perception

sj-txt-2-ipe-10.1177_20416695251364725 - Supplemental material for Perceiving direction of deformation-based motion Supplemental material, sj-txt-2-ipe-10.1177_20416695251364725 for Perceiving direction of deformation-based motion by Takahiro Kawabe in i-Perception

sj-txt-3-ipe-10.1177_20416695251364725 - Supplemental material for Perceiving direction of deformation-based motion Supplemental material, sj-txt-3-ipe-10.1177_20416695251364725 for Perceiving direction of deformation-based motion by Takahiro Kawabe in i-Perception

sj-txt-4-ipe-10.1177_20416695251364725 - Supplemental material for Perceiving direction of deformation-based motion Supplemental material, sj-txt-4-ipe-10.1177_20416695251364725 for Perceiving direction of deformation-based motion by Takahiro Kawabe in i-Perception

sj-txt-5-ipe-10.1177_20416695251364725 - Supplemental material for Perceiving direction of deformation-based motion Supplemental material, sj-txt-5-ipe-10.1177_20416695251364725 for Perceiving direction of deformation-based motion by Takahiro Kawabe in i-Perception
